# Shape memory polymer ovarian vein embolisation in a patient with nickel allergy

**DOI:** 10.1186/s42155-021-00212-y

**Published:** 2021-03-02

**Authors:** Davide Castellano, Andrea Boghi, Luca Di Maggio, Alessandro Rapellino, Daniele Savio

**Affiliations:** Department of Interventional Radiology and Neuroradiology, San Giovanni Bosco Hospital, Piazza del Donatore di Sangue, 3, 10154 Turin, Italy

**Keywords:** Shape memory polymer, Vascular plug, Peripheral embolisation, Pelvic congestion syndrome, Nickel allergy

## Abstract

**Background:**

Ovarian vein embolization in pelvic varicocele is usually obtained using nitinol coils. These devices can not be used in patients with proven nickel allergy.

**Case presentation:**

Shape memory polymer is a new embolic material available to interventionalists. A patient presented with pelvic congestion syndrome requiring embolisation of the left ovarian vein. The target vessel consisted of two twisted branches, each 5–6 mm in diameter. The patient also had a known allergy to nickel. Considering the anatomy and allergy status, embolisation with polyurethane shape memory polymer vascular plugs was considered a good case strategy. The embolisation procedure was technically successful with the deployment of two shape memory polymer plugs into each of the two left ovarian vein branches. Follow-up magnetic resonance imaging at 4 months showed sustained occlusion of the treated vessels and the patient showed no signs of allergy to the implanted material.

**Conclusions:**

In conclusion, our case presented an opportunity to utilise a new embolic material and achieve a good outcome in a patient with an allergy that may have resulted in complications when using metallic implants.

## Introduction

Shape memory polymer is a material that can exist in two stable shapes. The polyurethane shape memory polymer that is part of the IMPEDE Embolization Plug family (Shape Memory Medical, Santa Clara, California, USA) is a porous embolic scaffold when in its expanded shape. The shape memory polymer is crimped during manufacturing of the devices and the crimped shape is compatible with standard catheter-based delivery into the vasculature. When the device is deployed into the warm and aqueous environment of a blood vessel, the polymer recovers, or remembers, its original shape and self-expands. The porous nature of the expanded vascular plug supports the acute formation of thrombus throughout its structure and preclinical work has demonstrated that increased cellular infiltration leads to advanced healing of the initial thrombus to mature collagenous connective tissue when compared to other common embolic devices (Jessen et al. 2020).

## Case

**Fig. 1:**
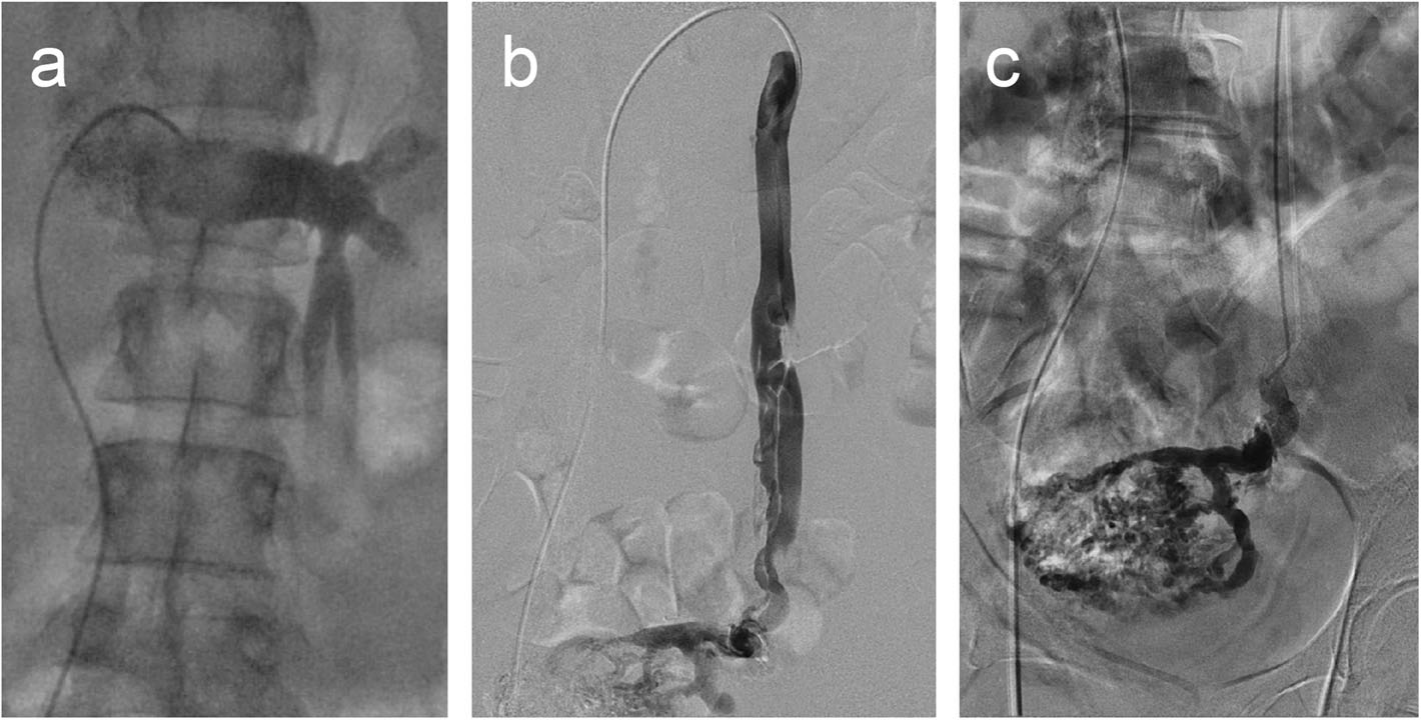
Pre-embolisation contrast injections (**a**-**c**) illustrating the anatomy and reflux in the target vessel and into the transpelvic varices. The left ovarian vein comprises of two twisted branches, each 5-6mm in diameter

**Fig. 2:**
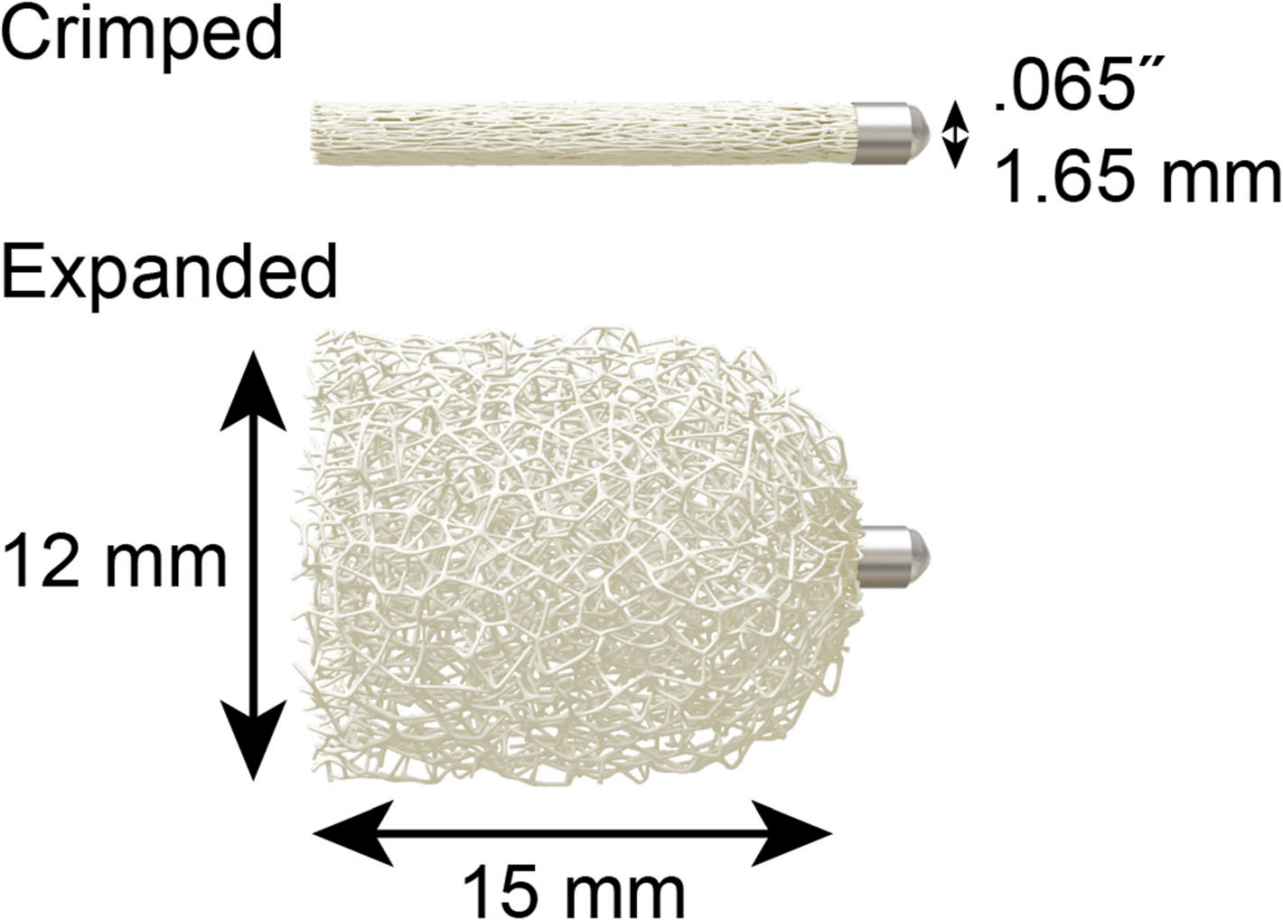
Illustrative structure and dimensions of the IMPEDE-FX Embolization Plug used in this case (IMP-FX-12)

A 35-year-old female presented with constant pelvic pain suggesting pelvic congestion syndrome. Magnetic resonance imaging revealed dilation of the left ovarian vein and vessel embolisation was indicated. Figure 1(a-c) shows the anatomy of the left ovarian vein upon catheter-based venography, i.e., two twisted branches, each 5–6 mm in diameter. The patient also had a nickel allergy discovered through allergy tests for skin reactions after wearing bracelets and rings. While rare, reactions to implanted devices in patients with metal allergies (including nickel) exist in the literature (Fahrni et al. 2015; Stamm et al. 2017; Clague et al. 2012). Furthermore, the metallic content of endovascular devices is not always apparent (Univers et al. 2018). Therefore, the use of a shape memory polymer vascular plug was considered a reasonable strategy to minimise the potential of an allergic reaction to implanted metallic embolic material in a patient with a known hypersensitivity, and especially in an ovarian vein case, where a large amount of embolic material is often implanted. The IMPEDE-FX Embolization Plug is comprised solely of a shape memory polymer plug and a platinum/iridium proximal marker. Figure2 shows the dimensions of the plug used in this case (IMP-FX-12). The volume of the shape memory polymer material in this size is approximately 1.25 mL. The left ovarian vein was accessed with a 90 cm 5 Fr Flexor Ansel Guiding Sheath (Cook Medical, Bloomington, Indiana, USA) and a 0.035 Radifocus Guidewire M (Terumo Corporation, Tokyo, Japan) using standard techniques via the right femoral and left renal vein. The first ovarian vein branch was accessed and the first IMPEDE-FX Embolization Plug was pushed into the vessel just proximal the vessel twist. The twist in the vessel branch minimised the likelihood of reflux-induced distal (caudad) device migration while the shape memory polymer was undergoing initial expansion. The catheter was retracted 3–4 cm from the device to allow blood flow into the vascular plug structure and to deliver contrast injections without putting unnecessary pressure on the vessel. Holding the catheter in place also mitigated any potential proximal (cephalad) migration risk, although this was not observed. Approximately 10 minutes later, and after initial expansion of the first plug was confirmed by contrast injection, a second shape memory polymer plug was deployed into the same branch just proximal to the first. Again, expansion was confirmed prior to removing the catheter in order to mitigate any potential proximal migration risk. The other branch was accessed and two more shape memory polymer plugs were deployed with a similar technique.

**Fig. 3:**
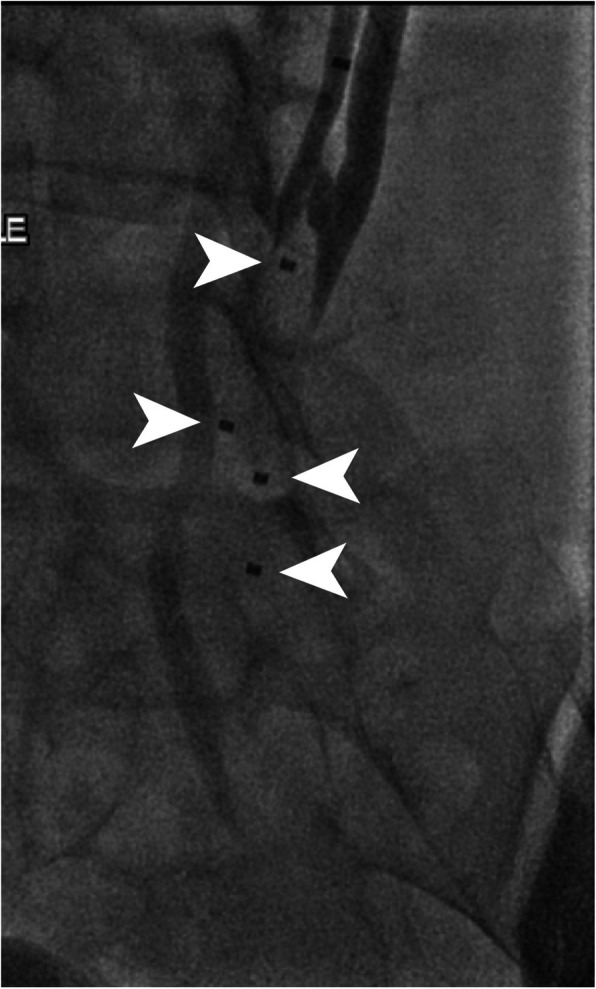
Final case image showing acute occlusion of the vessels with two IMPEDE-FX Embolization Plugs in each branch. Only the proximal marker is radiopaque (arrows). The radiolucent shape memory polymer portions of the devices are distal (caudad) to the radiopaque markers

It was not necessary to embolise the trans-pelvic varices with sclerosant. No other embolic material was used in the treated vessels. Ten minutes after deployment of the fourth plug, plug expansion was confirmed by contrast injection and the case was finished using standard techniques. Figure 3 shows the final case image. Follow-up magnetic resonance imaging at 4 months post procedure showed a sustained occlusion of the treated vessels and the patient showed no signs of allergy to the implanted material.

## Discussion

The features of this case presented an opportunity to take advantage of a new embolic material. The patient’s nickel allergy raised concerns about employing metallic implants and therefore, a novel polymer-based option was considered a good strategy. The twisted vessel branches in the low pressure venous system and ability to significantly oversize the polymeric implant minimised the potential of migration. The radial force of the expanded shape memory polymer is low and therefore inserting a 12 mm diameter device in a 5–6 mm vessel did not cause significant concern (Landsman TL et al. 2016). No significant distension of the vein around the implants was observed. One would normally consider placing the IMPEDE-FX Embolization Plug behind a device with some anchoring functionality (e.g., an IMPEDE Embolization Plug or other coil) but the anatomy enabled us to employ the IMPEDE-FX Embolization Plugs alone and to thereby minimise the amount of metallic implant. Furthermore, we took the time to confirm the expansion of each device prior to the next phase of the case. The catheter was retracted 3–4 cm proximal to the devices during their expansion to mitigate proximal migration risk and to enable easy confirmation of flow cessation via contrast injection. The shape memory polymer devices have a working time of 1 minute, i.e., they should be delivered into the target vessel within 1 minute of being pushed into the catheter. The devices start to slowly expand when in the catheter because they are in a warm and aqueous environment and if too much time is taken during delivery, friction may be encountered. We did not flush the devices prior to insertion (which must be included in the working time, if flushed) and delivered the devices well within the designated working time. We chose our catheter and guidewire based on manufacturer recommendations – the catheter had an internal diameter of .074”, which accommodated the device and no delivery friction was encountered.

Magnetic resonance imaging follow-up showed sustained occlusion of the target vessel. Another feature of the shape memory polymer devices is their limited artifact upon intraprocedural and follow-up imaging. This is particularly advantageous when trying to visualize complex anatomy such as the twisted vessels in our case, i.e., it is easy to see vessels that are behind the radiolucent shape memory polymer portion of the devices.

## Conclusions

In conclusion, our case presented an opportunity to utilise a new embolic material and achieve a good outcome in a patient with an allergy that may have resulted in complications when using metallic implants.

## Data Availability

Not applicable.
